# Videofeedback-guided teleintervention for the family of a child with a cochlear implant: a case study

**DOI:** 10.1590/2317-1782/20232022231en

**Published:** 2023-11-20

**Authors:** Ingrid Rafaella Dantas dos Santos, Wanderson Laerte de Oliveira Carvalho, Joseli Soares Brazorotto

**Affiliations:** 1 Programa Associado de Pós-graduação em Fonoaudiologia, Departamento de Fonoaudiologia e Laboratório de Inovação Tecnológica em Saúde, Universidade Federal do Rio Grande do Norte – UFRN - Natal (RN), Brasil.; 2 Departamento de Matemática e Estatística, Universidade do Estado do Rio Grande do Norte - Mossoró (RN), Brasil.

**Keywords:** Correction of Hearing Impairment, Audiovisual Aids, Formative Feedback, Remote Consultation, Family, Case Report

## Abstract

Interventions for parental training for families of hard of hearing children, including cochlear implant users, are identified as optimizing their developmental outcomes. In this single-case intervention study, we aim to describe the use of videofeedback in a remote environment, as well as to identify its effectiveness, based on the analysis of mother-child interaction, both for the mother's communicative behaviors and for the behaviors of the mother, receptive and expressive language of the child. Pre- and post-intervention measurements were performed, based on video analysis of the mother's interaction with the child, by blind judges, as well as through the application of assessment instruments for the child and the mother. There were 13 sessions, 3 of which were for evaluation before and after the intervention and 10 of teleconsultation sessions in which the videofeedback tool was used with the mother. Data were analyzed descriptively and inferentially, using the JT method, which determined the Reliable Change Index (BMI) and Clinical Significance. There was reliable positive change in the child's receptive and expressive language, as well as reliable positive change and clinically significant change in mother-child interaction after the 10 sessions of remote videofeedback intervention. Based on the reliable changes observed in this study, we present this model (televideofeedback) as a potential to optimize resources and efforts for therapeutic success in children's auditory rehabilitation, which should be studied in research with a rigorous method, for the broad recommendation of its use.

## INTRODUCTION

The complex and multifaceted interactional processes that take place between the children and their caregivers are considered vital for healthy development^(1)^, providing or limiting opportunities for stimulating the child’s hearing, language, and social aspects, which are acquired through effective and synchronized interaction^(2)^. The use of parenting interventions after the diagnosis of hearing loss makes caregivers, including family members and anyone regularly involved in the child's care, more active and responsive in communicating with children. However, parental support provides an understanding of the roles of caregivers, both in establishing the consistent use of hearing aids and in optimizing the child's hearing and language development, essential aspects for effective results in this population^(3-7)^.

Thus, based on the premise that the family environment plays an important role in promoting the development of oral language skills in children with hearing loss^(8)^, it is worth considering the different models proposed for family-centered interventions, to increase the level of support and enrichment of family interactions, with a potential impact on the psychosocial and neurocognitive development of children with hearing loss.

Therefore, research dedicated to studying different models of intervention with families can help the speech therapist understand which program pattern best suits the context and needs of the assisted family, varying its intensity, duration, framework (individual, group, or mixed), as well as where it can be implemented (at home, in the clinic or service or remotely)^(2-7)^.

Among the models of parental training or intervention with families, there are those focused on the interactions between caregivers and hard of hearing children (users of different types of devices, including the Cochlear Implant), which have been consistently related to enhancing the quality of the linguistic stimuli offered, also giving families more confidence to deal with the daily situations they experience. Thus, facilitating the listening and language environment for their children, shaping an ideal communicative scenario that encourages the child's language initiative and autonomy, especially in the first years of life, to take advantage of the window of opportunity for development^(2-7)^.

To work with interaction, the techniques of recording videos and therefore analyzing the interaction of the family-child dyad have been used over the years, initially by Psychology, as in the primary studies of Harry Biemans (1990) based on Trevarthen's theory of intersubjectivity^( (2))^, later introduced in the United Kingdom, spreading rapidly in clinical practice, with the aim of working on the development of attuned interactions. Recently, video analysis of family-child interaction has been used more systematically in studies of children's auditory rehabilitation^(2,5,6)^.

The use of the video feedback (VF) procedure^(2)^ offers a possibility of intervention that makes it possible to improve the quality of the family-child relationship by reinforcing the positive interactions that exist in the video analyzed. VF is an intervention model that involves changes in individuals to improve relationships and/or communication in a variety of contexts, including clinical and at-risk populations such as families and children with hearing loss, promoting a positive change that is favorable to child development and family-child interaction^(2,5,6)^.

In this way, by viewing positive clips of the interaction between the adult and the child, reflections are made with the family about their potential as a stimulating agent. There is also guidance on the aspects that can be improved in the child's hearing and language environment, generating a positive cycle of empowerment and training for parents to interact with their hard of hearing children^(5,6)^.

As its basis is the recording and analysis of the video by the professional, followed by the VF session with the family, during the Sars-Cov-2 pandemic, its use in remote format began to be considered. Thus, given the possibility of synchronous and asynchronous teleconsultations under the regulations of the Federal Council of Speech and Hearing Therapy (CFFa), CFFa resolution no. 580/2020^(9)^ in Brazil, this research was undertaken to investigate its feasibility based on the application of a clinical case in a pilot study.

It should be noted that remote intervention models with hard of hearing children have already been studied and have been highlighted as effective for children's hearing rehabilitation. This is especially because it optimizes the participation of the family as the protagonists of changes in their children's development, as well as financial and logistical benefits in terms of access to trained professionals^(7)^.

Based on these considerations, the question of this study was: “Is televideofeedback, a low-cost technological tool, applicable and does it promote change in communication behaviors in the mother-child dyad studied?”. The hypothesis is that this model can be used by the speech therapist in teleconsultations and can be a catalyst for a more attuned interaction between the family and the child, with benefits for their communication.

Considering the statistics of hearing loss^(10)^ in the world and in Brazil and the impossibility of having enough specialist professionals in all regions of the country, as well as the need to provide families with individualized support and specific information to improve the development of children with hearing loss, based on their daily interactions, this study aimed to describe, through a single-case intervention study, the use of the VF tool, applied in a remote environment, as well as to identify the reliable changes in the interaction between mother and child and in the receptive and expressive language of the child, a cochlear implant user, before and after the intervention.

## CLINICAL CASE PRESENTATION

### Study design

This is a single-case intervention study, approved by the institutional Research Ethics Committee (CAAE ‒ 13453319.0.0000.5292, under Report No. 3.440.683). The participation of the mother, the child's main caregiver, was conditional on accepting the invitation, as well as signing the Informed Consent Form (ICF) and the Image Form (Voice/Video), in digital format. The writing of this case study was based on the CARE checklist (*Checklist of information to include when writing a case report*).

A total of 13 teleconsultation sessions were carried out, three of which were evaluations (two pre-intervention and one post-intervention) and ten intervention sessions, intensively. Each session lasted 50 minutes, occurred on five weekdays, and was distributed over two weeks.

### Inclusion and exclusion criteria

As inclusion criteria for inviting the participating family, the child should have a diagnosis of hearing loss of any type or degree, with no other disabilities associated with the hearing impairment, make effective use of an Individual Sound Amplification Device (ISAD), and/or Cochlear Implant (CI), be up to six years old, and be undergoing rehabilitation in the Hearing and Language program at the SUVAG/RN Center, with attendance equal to or greater than 75%. In addition to being willing to take part in the intensive intervention, which took place over 10 consecutive days, excluding weekends, the family had to have an electronic device (computer, tablet, or smartphone) with internet access that allowed them to connect. Inclusion criteria were established, focusing on the population of hearing families. Thus, if the main guardian was deaf, they would not be included in the study, although the same intervention protocol could be applied to deaf families, provided the researcher was fluent in Brazilian Sign Language (LIBRAS).

The exclusion criteria were a child who had not yet adapted to ISAD or CI, used LIBRAS, had an attendance rate of less than 75%, and had other disabilities associated with hearing loss, so we decided to study a case without other disabilities associated with hearing loss. However, it should be noted that the proposed intervention is applicable to specific groups.

Regarding the family, their unwillingness to take part in the intensive intervention and their inability to access the internet were exclusion criteria in this study. The characterization of the child and their mother is shown in Tables 1 and 2.

**Table 1:** Characterization of the child participating in the research

**Child**	**Age (y/m)**	**Gender**	**HA (years/months)**	**Grade of SNHL**		**Etiology of HL**	**Adaptation**	**Device type**		**Categories of Hearing and Language**	
				**RE**	**LE**			**RE**	**LE**	**HC**	**LC**
C0	5y2m	female	3y5m	Profound	Profound	Idiopathic	Bilateral	CI	TA	5	4

**Source:** own authorship

**Caption:** C0 = Child 0; y/m = years and months; HA = Hearing age; HL = Hearing Loss; SNHL = Sensorineural Hearing Loss according to WHO 2014; RE = Right ear; LE = Left ear; CI = Cochlear Implant; TA = Cochlear Implant in Technical Assistance at the time of the intervention; HC = Hearing Category; LC = Language Category^(11)^.

**Table 2:** Characterization of the family participating in the research

**Family**	**Age (y/m)**	**EL**	**Occupation**	**Income**	**Aid**	**FER**		**RSE**		**PSS**	
F0	34y9m	CHS	Manicure	1 minimum wage	does not have	65	67	33	37	26	24

**Source:** own authorship

**Caption:** F0 = Family 0; y/m = years and months; EL = Education Level; CHS = Completed high school; FER = Family Environment Resources; RSE = Rosenberg Self-Esteem Scale; PSS = Parental Stress Scale

### Case characterization and evaluation tools

For the child's assessment, which was carried out remotely, it was used the Hearing and Language Categories^(11)^ whose scores were obtained from the observation of the speech therapist in charge of the service's rehabilitation team and the Brazilian Functional Hearing Performance Indicators - Reduced Version (FAPI-r) family form was applied. The reduced version of the FAPI-r consists of a 25-item form for the speech therapist to use with the child and a 15-item form for the family. Both are organized into 15 sections in which the family member reports the frequency with which auditory behaviors are observed at home, covering the skills of sound awareness, auditory feedback, auditory discrimination and recognition, auditory comprehension, auditory short-term memory, and auditory linguistic processing^(12)^.

In the application of the FAPI-r (family version), it was observed that at the pre-intervention moment, according to the mother's observations, the child had already acquired the skills of sound awareness (100%), auditory feedback (100%) and was in the process of acquiring the skills of auditory discrimination and recognition (75%), auditory comprehension (75%), auditory short-term memory (66,7%) and auditory linguistic processing (75%). At the post-intervention stage, there was a change in the scores for auditory discrimination and recognition skills (100%) and auditory comprehension (100%). The others remained with the same scores. Although it cannot be said that this change was the result of the intervention process, it was observed that the questions in which the mother reported an improvement in her hearing skills referred to: discrimination of the communicative intent of statements, identification of critical elements in sentences (two and three elements)and in a children's story, indicating that the dialogic process enriched by the proposed intervention may have influenced the mother's perception and her greater attention to these skills during interactions with her daughter.

The following protocols were used to assess the mother:

1. Parental Stress Scale (PSS) - This is a self-report instrument with 18 items representing four factors (parental rewards, parental stressors, lack of control, and parental satisfaction) answered on a Likert scale (zero to five) designed to measure the level of stress experienced by fathers and mothers of children under the age of 18. The total score can vary between 0 and 72 points, so the higher the score, the greater the parental stress^(13)^.
2. Rosenberg Self-Esteem Scale (RSE) - an instrument with ten closed sentences which aims to provide an overall assessment of the individual's positive or negative attitude towards themselves, answered on a Likert scale (one to four), ranging from “totally agree” to “totally disagree”. The score can oscillate between 10 and 40 and the higher the score obtained on the scale, the higher the individual's level of self-esteem^(14)^.
3. Family Environment Resource Inventory (FER): script applied in the form of a semi-structured interview to characterize material resources and family routines. The sum of the scores for items 1 to 7 results in a gross score, while topics 8, 9, and 10 have specific scores indicated on the form^(15)^.

It is worth noting that although these protocols were applied to characterize and monitor the mother, in this pilot study we did not aim to verify the effect of the intervention on the measures of family environment resources, parental stress, and the mother's self-esteem, considering the specific characteristics of the intervention, its short term and the complexity of the variables analyzed.

It was observed that in the assessment of parental stress, comparing the pre- and post-intervention scores, there was a slight decrease in the score, but in the pre-intervention assessment there were already low levels of parental stress.

The RSE, measured by the total of the items, is classified into self-esteem categories: low, medium, and high. Since the pre-intervention assessment, the mother had high self-esteem, scoring above 30 in both scenarios.

It should be noted that this was a case in which the mother's characteristics (schooling, parental stress, and self-esteem) were positive for the therapeutic work. Therefore, it is important that these measures are considered when assessing families to better understand the needs of each one of them so that more assertive approaches can be adopted.

Regarding the family environment, the FER showed a view of the resources, characteristics, and behaviors at home, with a slight increase in the examples given by the mother. She described the closeness of the family in carrying out shared activities inside and outside the home environment on a regular basis.

A change was noticed in the examples of item 4, where pre-intervention the mother listed “*playing, watching movies, watching children's programs on TV, reading books, magazines*” and after the intervention she changed the items to “*playing, watching movies, listening to the child's stories; talking about the subjects she brings up*”.

In item 8, the mother referred to herself in all the situations, as not only the main caregiver, but also an agent who conducts stimulating activities.

The availability of physical resources such as toys, games, magazines and books remained the same before and after the intervention.

According to the mother's account, her attention was refocused post-intervention, to listen more to what the child brings to the daily routine, as well as the perception of her empowerment as an agent who cooperates with her daughter's learning, based on daily activities.

In addition to these evaluations, all 11 interaction videos recorded between January 9 and 21, 2022 (excluding weekends) were analyzed using the Family-Child Interaction Analysis Tool (adapted by the researchers from Cole^(16)^) by three independent judges, two of whom were blind.

The judges have a degree in speech therapy, more than four years' experience in auditory rehabilitation and familiarity with using the tool in clinical practice.

The Family-Child Interaction Analysis Tool consists of an observational scale of communicative behaviors in the interaction between hearing parents and their hard of hearing children. The frequency of behaviors is scored on a Likert-type scale, from 1 to 7, with the lowest frequency being “I haven't seen” and the highest frequency being “I've always seen”. There are also “not sure” or “not applicable” fields in the emotional and behavioral dimensions of the child and the main caregiver.

The instrument also contains a general analysis of the caregiver's communicative competence, scored on a scale of 0 (shows no competence), 1 (shows a little competence), 2 (shows good competence) or 3 (shows excellent competence).

Agreement between the judges was almost perfect, with Kappa of 0.79 and 91% agreement (p ≤ 0.007), as shown in Table 3.

**Table 3:** Agreement between the judges

**Method**	**Cohen's Kappa**
Videos	11
Judges	2
Agreement %	91
Kappa	0.792
z	2.69
p-value	0.007

**Source:** own authorship

### Interventions concomitant with the study

Alongside the VF tele intervention sessions of this study, the children and their mothers continued to attend the group and individual sessions offered by the Hearing and Language program. In this context, with remote participation due to the pandemic, they have participated in two ways. Firstly, they attended individual speech therapy sessions with the family and the child once a week according to the Aurioral Method. Secondly, they had group sessions three times a week, two focusing on auditory stimulation and one on family needs. Each session lasted 30 minutes.

### Videofeedback teleintervention procedures

The VF teleintervention was based on a model already tested with families of hard of hearing children^(5,6)^ and was carried out in 10 synchronous 50-minute sessions, with an individual framework.

To carry out the procedures with the family, the researcher used a computer with a webcam and headphones and a wired fiber optic broadband internet connection with a connection speed of 400 megabytes. The teleconsultation environment followed all the rules of the resolution of the Brazilian Federal Council of Speech and Hearing Therapy (CFFa) No. 580/2020(9), carried out on a secure platform, in a confidential manner.

For the teleintervention to be operational, the mother had to be trained beforehand to familiarize her with the GSuite video call platform, as well as preparing for the recording and uploading of the videos via GSuite Drive, which comprised the first of the 13 sessions.

The interaction videos were recorded by the mother herself, on her own cell phone, with an average duration of ten minutes per video, and then shared with the researcher via cloud storage. The VF session was scheduled after the sending signal.

It's important to note that the environment in which the videos were recorded was natural, familiar to the child, and with the resources available in the child's own home. The mother was instructed to interact with the child in natural situations at home, such as her routine.

The main researcher analyzed each video of the interaction and proceeded to plan the sessions, always highlighting 3 positive points observed in the interaction and preparing guidelines to improve the mother's communicative interaction with her daughter.

The interaction video with the selection of positive micro-moments was shown to the mother via Google Meet's screen sharing function. During the sessions, which took place in a dialogical manner, the mother was led to reflect on the clip presented and the positive points of each clip were discussed, and guidance given to improve communication.

All the data analyzed was entered into an Excel® spreadsheet for descriptive and qualitative analysis, based on the observations made at the end of the adapted protocol^(16)^.

The inferential analysis of the data presented in this manuscript was carried out using the JT statistical method^(17)^, which makes it possible to analyze reliable change and clinical significance by verifying the reliability of the changes obtained between the pre- and post-intervention scores, with the aim of identifying whether the changes, even in studies with only one or a few participants, are of a reliable nature and whether they are clinically relevant, investigating the Reliable Change Index - RCI and the Clinical Significance of the changes.

The results presented below comprise the analysis of the videos carried out by the blind judges, highlighting the variables of the mother's general communicative competence and the child's receptive and expressive language behaviors.

### Results

**Figure 1:**
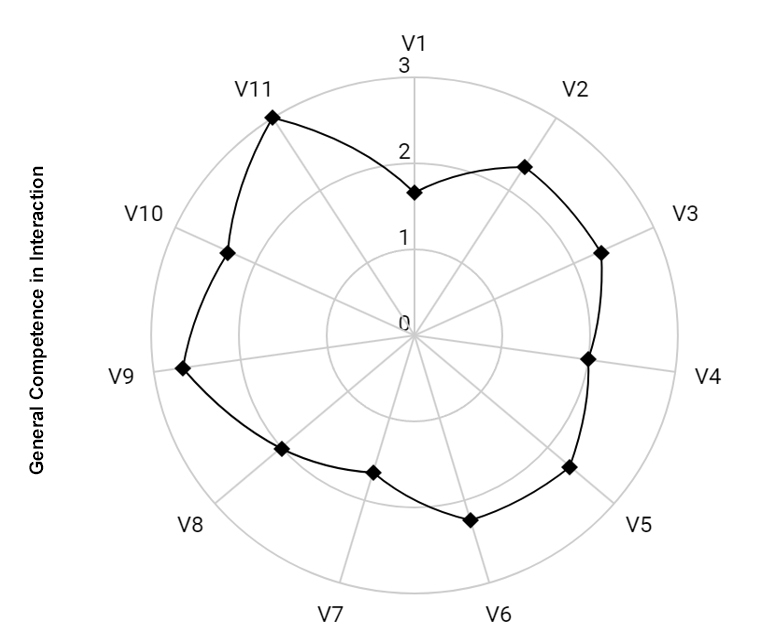
Mother's General Communicative Performance throughout the video feedback-guided teleintervention process. **Source:** own authorship

**Figure 2:**
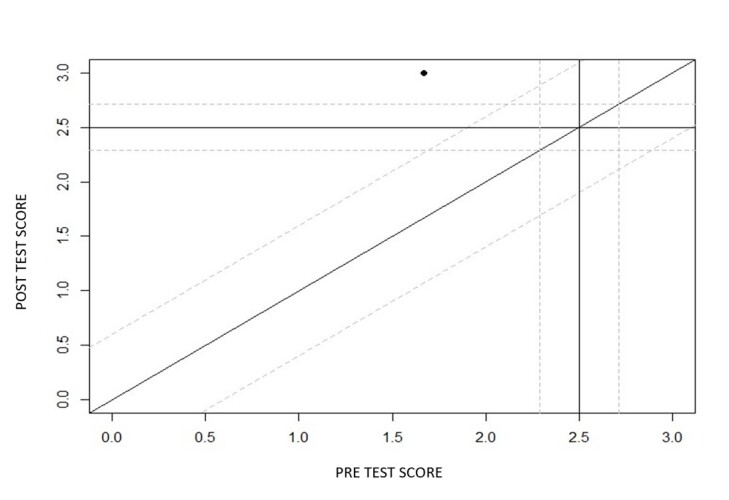
Reliable change index of the variable “Mother's General Competence in Interaction” before and after video feedback-guided teleintervention sessions. **Source:** own authorship

Regarding the intervention process, it was noted that the mother's general communication skills improved over the course of the sessions. She went from scores of 1 to 2 (from low competence to good competence) and until the eighth video she remained between scores 2 and 3 (except for video 7, where a slight drop in her performance in the interaction was noted). From the ninth video onwards, the mother, who had scores of good communicative competence (2), was evaluated by the two judges and the researcher as having excellent overall communication competence during the interaction. This indicates that 8 sessions were necessary for the mother to reach the maximum level of performance in the interaction according to the analysis tool used, as shown in Figures 1 and 2.

**Caption:** V1 a V11: Videos 1 to 11. 0: does not show communicative competence; 1: shows poor communication competence; 2: shows good communicative competence; 3: shows excellent communication competence.

**Figure 3:**
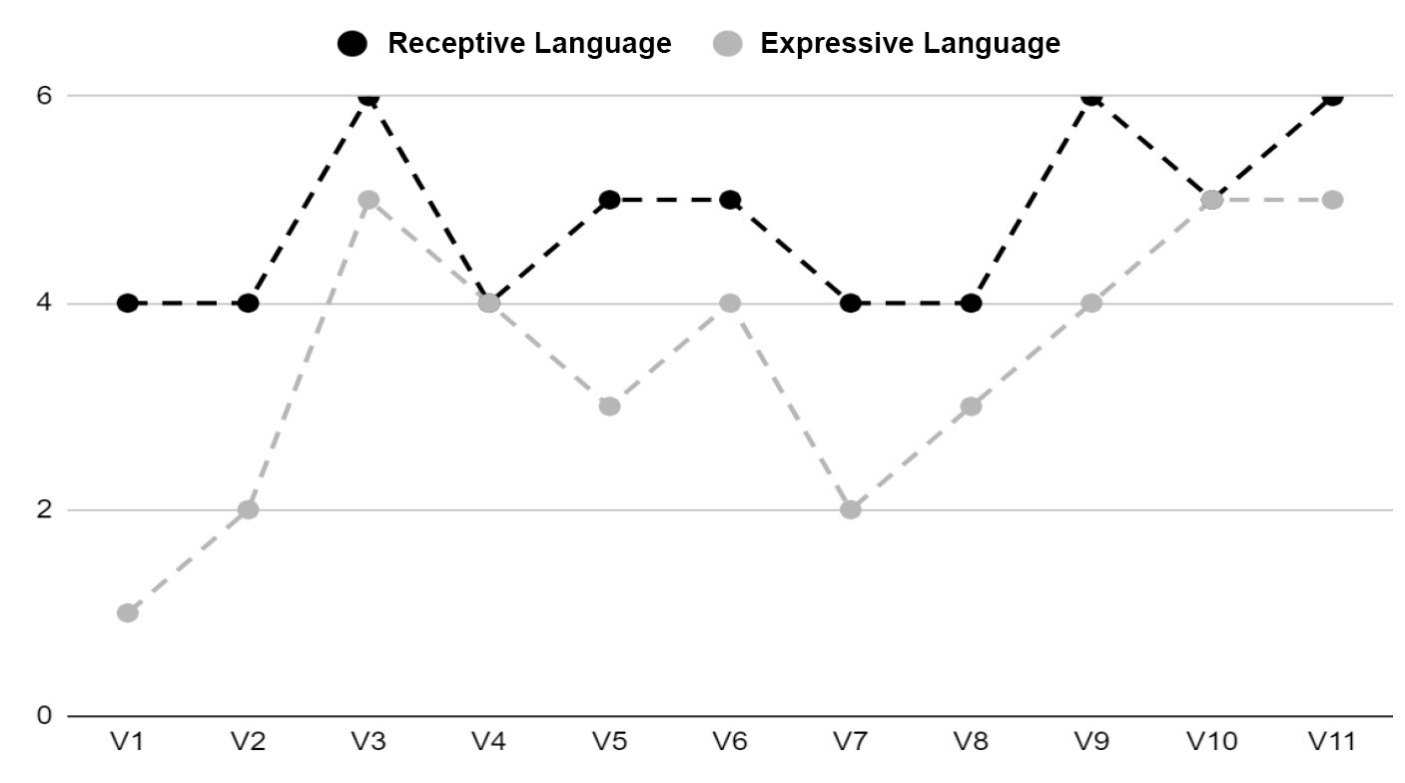
Children's Behaviors: Receptive and Expressive Language. **Source:** own authorship

**Figure 4:**
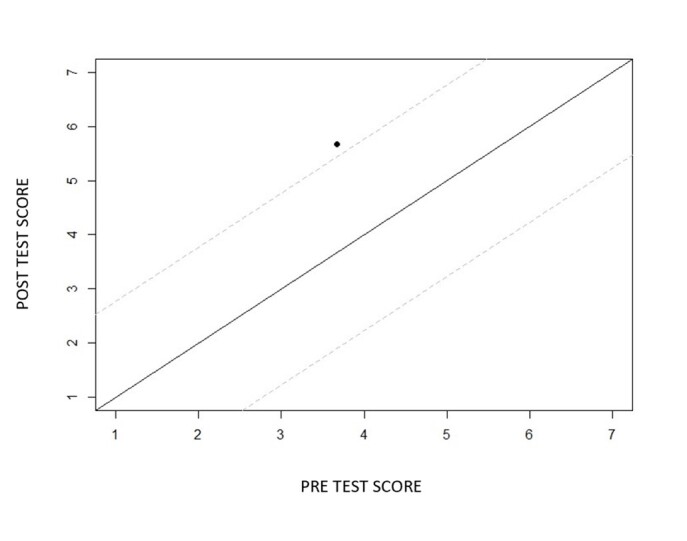
Reliable change index of the Receptive Language variable before and after video feedback-guided teleintervention sessions. **Source:** own authorship

**Figure 5:**
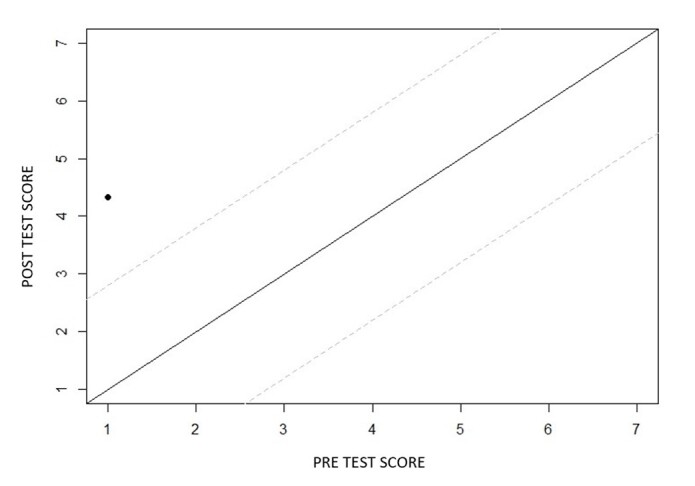
Reliable change index of the Expressive Language variable before and after video feedback-guided teleintervention sessions. **Source:** own authorship

As for the child's performance, as shown in Figures 3, 4, and 5, there was an improvement in receptive and expressive language, and the intervention produced a reliable positive change (above the confidence interval) for the variables studied in the child's behavior: “Receptive Vocabulary” “Expressive Vocabulary”.

## DISCUSSION

The communicative competence of the main caregiver of a child with hearing loss has been shown to be an important factor in their hearing and language development^(8)^. Therefore, interventions that optimize the dialogical interaction between families and caregivers with these children are important and can be implemented in children's hearing rehabilitation services, preferably at an early stage^(2-7)^.

In our case study, we observed that there was a progressive improvement in the mother's communication skills over the course of the sessions and that from the eighth session onwards (ninth video) there was a significant qualitative leap in this variable. Through the JT Method^(17)^, it was possible to simultaneously demonstrate the reliability of the changes and the clinical significance in this case observed in the mother (Figure 2).

Thus, the mother showed different scores pre- and post-intervention, achieving a performance compatible with that of a non-clinical population, falling within the “recovery” classification suggested by the authors^(17)^ when both criteria are reached. This data corroborates the literature on the potential of the VF tool to improve communicative behavior and interaction between hearing caregivers and their hard of hearing children^(2,5,6)^.

It was observed during the teleconsultations that allowing the mother to be the child's stimulating agent directly, even under the remote guidance of the speech therapist, promoted an important and necessary change in her role about her daughter, which is confirmed by studies that have used teleaudiology in the care of families of hard of hearing children^(7)^.

Thus, considering the number of hours the deaf or hard of hearing child is regularly at home or in school, using methods that direct the child's families and caregivers' attention to optimize their daily interactions is justified. It becomes an agent for optimizing these children's daily learning possibilities^(2-8)^.

Regarding the effect of parental interventions on the language development of hard of hearing children, some studies point to significant improvements^(2-4)^. However, more robust evidence is needed to state whether the effect of interventions on families is maintained over time, as well as their impact on the child's performance longitudinally.

For the child participating in this study, based on the analysis of the Reliable Change Index (RCI)^(17)^, calculated using the values of the evaluation instrument applied, was considered a reliable and positive index when the values reached numbers higher than 1.96, so we can infer that the intervention produced a reliable positive change (above the confidence interval) for the variables studied in the child's behavior: “Receptive Vocabulary” (Figure 4) and “Expressive Vocabulary” (Figure 5).

However, there was no clinical significance for either measure, i.e. there was an improvement in the child's receptive and expressive language behaviors, but not enough to place them in the non-clinical population. This was to be expected, as the language development needs of hard of hearing children are extensive^(8)^ and 10 sessions of indirect therapy (focused on training the mother) would not be expected to be sufficient to meet all the specific speech therapy intervention needs for the child's language development. Even so, there was reliable improvement (reliable positive change), and the participant was categorized as “improved” (achieved RCI but not clinical significance)^(17)^.

As it was not a controlled clinical environment, some variables were noticed during the sessions, such as the lack of interference from other family members, external noises, and difficulty in positioning the camera. On the other hand, we observed spontaneity in the interaction between mother and child, the availability of material resources from the routine and the child's comfortable environment in a non-clinical situation.

By analyzing the child's and adult's attempts to communicate and the exchange of conversational turns, you can understand the child's real linguistic environment. This analysis, also made possible by technological resources such as LENA (Language Environment Analysis System)^(8)^, can improve interventions such as video feedback-guided teleintervention.

Based on this case study, there is a need to use this methodology in randomized experimental studies with an adequate sample size to estimate the effects of this parental training model, to optimize efforts for therapeutic success in the population of hard of hearing children.

## FINAL COMMENTS

Given the scarcity of research on VF programs and their applicability in hearing rehabilitation, this study is relevant because it contributes to the production of evidence on the effectiveness of VF teleintervention and expands the possibility of implementing programs using this tool.

Although this was a single case, the analysis using the Jacobson and Truax (JT) method allowed us to verify the effectiveness of the intervention, measured by the reliable change in the mother's interaction behaviors with her daughter and the child's receptive and expressive language, with a significant clinical change in the mother's communicative behaviors.

As this is a single case study, it is not yet possible to generalize these results. Further research with greater methodological robustness and investigation into the guarantees of maintaining results with the intervention is needed, which is the main limitation of this study.

The continuity of research into parental training for families of children with hearing loss is relevant in Brazil, given the diversity of our culture and the situations of vulnerability that may have repercussions on the need to develop specific training models for our population.
