# Supplementary material for: Videofeedback-guided teleintervention for the family of a child with a cochlear implant: a case study
Source: Codas. 2023 Nov 20;35(6):e20220231. doi: 10.1590/2317-1782/20232022231en (PMC10702711; doi:10.1590/2317-1782/20232022231en)
Supplement: Translation

# Translation: Teleintervenção guiada por videofeedback à família de uma criança usuária de implante coclear: estudo de caso

**DOI:** 10.1590/2317-1782/20232022231pt

**Authors:** Ingrid Rafaella Dantas dos Santos, Wanderson Laerte de Oliveira Carvalho, Joseli Soares Brazorotto

**Affiliations:** 1 Programa Associado de Pós-graduação em Fonoaudiologia, Departamento de Fonoaudiologia e Laboratório de Inovação Tecnológica em Saúde, Universidade Federal do Rio Grande do Norte – UFRN - Natal (RN), Brasil.; 2 Departamento de Matemática e Estatística, Universidade do Estado do Rio Grande do Norte - Mossoró (RN), Brasil.

**Keywords:** Correção de Deficiência Auditiva, Recursos Audiovisuais, Feedback Formativo, Teleconsulta, Família, Estudo de Caso

## Abstract

Intervenções para a capacitação parental de famílias de crianças com deficiência auditiva, incluindo as usuárias de implante coclear, são apontadas como otimizadoras em seus resultados de desenvolvimento. Neste estudo de intervenção de caso único, temos por objetivos descrever o uso do videofeedback em ambiente remoto, bem como identificar a sua efetividade, a partir da análise da interação da mãe e criança, tanto para os comportamentos comunicativos da mãe como para os comportamentos da linguagem receptiva e expressiva da criança. Medidas pré e pós-intervenção foram realizadas, a partir da análise de vídeos de interação da mãe com a criança, por juízes cegos, bem como pela aplicação de instrumentos de avaliação da criança e da mãe. Foram realizadas 13 sessões, sendo 3 de avaliação nos momentos pré e pós-intervenção e 10 sessões de teleconsulta em que se empregou a ferramenta de videofeedback com a mãe. Os dados foram analisados de forma descritiva e inferencial, por meio do método JT, que determinou o Índice de Mudança Confiável (IMC) e a Significância Clínica. Houve mudança positiva confiável na linguagem receptiva e expressiva da criança, bem como mudança positiva confiável e mudança clínica significativa na interação da mãe com a criança após as 10 sessões de intervenção remota com videofeedback. A partir das mudanças confiáveis observadas neste estudo, apresentamos este modelo (Televideofeedback), como potencial para otimizar os recursos e esforços para o sucesso terapêutico na reabilitação auditiva infantil, o qual deve ser estudado em pesquisas com método rigoroso, para a ampla recomendação de seu uso.

## INTRODUÇÃO

Os processos interacionais complexos e multifacetados que ocorrem entre as crianças e seus cuidadores são considerados vitais para um desenvolvimento saudável^(1)^, propiciando ou limitando as oportunidades de estimulação da audição, linguagem e dos aspectos sociais da criança, os quais são adquiridos por uma interação efetiva e sintonizada^(2)^.

A utilização de intervenções parentais após a confirmação do diagnóstico da deficiência auditiva torna os cuidadores, incluindo familiares e qualquer pessoa regularmente envolvida no cuidado da criança, mais ativos e responsivos na comunicação com as crianças. O suporte parental, portanto, proporciona o entendimento dos papéis dos cuidadores, sejam eles no estabelecimento do uso consistente do dispositivo auxiliar à audição, como também na otimização do desenvolvimento da audição e da linguagem da criança, aspectos essenciais para resultados efetivos nesta população^(3-7)^.

Assim, a partir da premissa de que o ambiente familiar desempenha importante papel na promoção do desenvolvimento de habilidades de linguagem oral em crianças com deficiência auditiva^(8)^, cabe considerar os diferentes modelos propostos para as intervenções centradas na família, a fim de elevar o nível de apoio e enriquecimento às interações familiares, com potencial impacto ao desenvolvimento psicossocial e neurocognitivo das crianças com deficiência auditiva.

Desta feita, pesquisas dedicadas a estudar diferentes modelos de intervenção junto às famílias podem auxiliar o fonoaudiólogo a entender qual o padrão de programa que mais se adeque ao contexto e às necessidades da família assistida, variando sua intensidade, duração, enquadre (individualmente, grupo ou misto), além do local onde pode ser implementado (domiciliar, na clínica ou serviço ou remoto)^(2-7)^.

Dentre os modelos de capacitação parental ou intervenção com famílias, há aqueles focalizados nas interações dos cuidadores e crianças com deficiência auditiva (usuárias de diferentes tipos de dispositivos, entre eles o Implante Coclear), os quais têm sido consistentemente relacionados à potencialização da qualidade dos estímulos linguísticos oferecidos, proporcionando também às famílias mais confiança para lidar com as situações diárias vivenciadas e, assim, facilitar o ambiente de audição e linguagem para seus filhos, moldando, desta forma, um cenário comunicativo ideal que encoraje a iniciativa de linguagem e autonomia da criança, principalmente nos primeiros anos de vida, de modo a aproveitar a janela de oportunidades para o desenvolvimento^(2-7)^.

Para trabalhar com a interação, as técnicas de registro de vídeos e conseguinte análise da interação da díade família-criança, têm sido utilizadas ao longo dos anos, inicialmente pela Psicologia, como nos estudos primários de Harry Biemans (1990) com base na teoria da intersubjetividade de Trevarthen^((2))^, posteriormente introduzida no Reino Unido, difundindo-se rapidamente na prática clínica, com intuito de se trabalhar no desenvolvimento de interações sintonizadas. Mais recentemente, as análises de vídeo da interação família-criança têm sido utilizadas em estudos na reabilitação auditiva infantil de maneira mais sistemática^(2,5,6)^.

A utilização do procedimento de *videofeedback* (VF)^(2)^ traz uma possibilidade de intervenção que viabiliza a melhora na qualidade da relação família-criança, com o reforço das interações positivas existentes no vídeo analisado. O VF é um modelo de intervenção que envolve mudanças dos indivíduos com propósito de aprimorar situações de relacionamento e/ou comunicação em contextos variados, dentre eles, populações clínicas e de risco como as famílias e crianças com deficiência auditiva, promovendo uma modificação positiva favorável ao desenvolvimento infantil e à interação família-criança^(2,5,6)^.

Desta forma, a partir da visualização de clipes positivos da interação entre o adulto e a criança são realizadas reflexões com a família sobre seu potencial como agente estimulador, bem como orientações sobre os aspectos que podem ser aperfeiçoados no ambiente de audição e linguagem da criança, gerando um ciclo positivo de empoderamento e capacitação dos pais para interação com seus filhos(as) com deficiência auditiva^(5,6)^.

Como sua base é o registro e análise do vídeo pelo profissional, seguida da sessão de VF com a família, por ocasião da pandemia do Sars-Cov-2, sua utilização em formato remoto passou a ser considerada e, no Brasil, dada a possibilidade da realização de teleconsultas síncronas e assíncronas sob regulamentação do Conselho Federal de Fonoaudiologia (CFFa), resolução CFFa nº 580/2020^(9)^, a referida pesquisa foi, então, iniciada de modo a se investigar, a partir da aplicação em um estudo piloto de um caso clínico, a sua viabilidade.

Ressalta-se que modelos de intervenção remoto com a população de crianças com deficiência auditiva já foram estudados e têm sido destacados como efetivos para a reabilitação auditiva infantil, especialmente porque otimizam a participação da família como protagonista das mudanças no desenvolvimento de seus filhos(as), além de benefícios financeiros e logísticos quanto ao acesso a profissionais capacitados^(7)^.

A partir destas considerações, a pergunta deste estudo foi: “o *televideofeedback*, ferramenta tecnológica de baixo custo, é aplicável e promove mudança nos comportamentos de comunicação na díade mãe-criança estudada?” A hipótese é de que este modelo é passível de uso pelo fonoaudiólogo em teleconsultas e pode ser um catalisador para a interação mais sintonizada entre a família e a criança, com benefícios para sua comunicação.

Considerando, pois, as estatísticas da deficiência auditiva^(10)^ no mundo e no Brasil e a inviabilidade de profissionais especialistas em número suficiente em todas as regiões do país, bem como a necessidade de se fornecer às famílias um suporte individualizado e com informações específicas para melhorar o desenvolvimento da criança com deficiência auditiva, a partir de suas interações diárias, este estudo teve por objetivos descrever, por meio de um estudo de intervenção de caso único, o uso da ferramenta de VF, aplicada em ambiente remoto, bem como identificar as mudanças confiáveis na interação da mãe e criança e na linguagem receptiva e expressiva da criança, usuária de implante coclear, antes e após a intervenção.

## APRESENTAÇÃO DO CASO CLÍNICO

### Tipo de estudo

Trata-se de um estudo de intervenção, de caso único, aprovado pelo Comitê de Ética em Pesquisa Institucional (n^o^ do parecer 3.440.683). A participação da mãe, cuidadora principal da criança, foi condicionada à aceitação do convite, bem como à assinatura do Termo de Consentimento Livre e Esclarecido (TCLE) e Termo de Imagem (Voz/Vídeo), em formato digital. A redação deste estudo de caso baseou-se no *checklist* CARE (*Checklist of information to include when writing a case report*).

Foram realizadas um total de 13 sessões de teleconsulta, sendo três de avaliação (duas no pré-intervenção e uma no pós) e 10 sessões de intervenção, de forma intensiva, com 50 minutos por sessão, nos cinco dias da semana, distribuídas, portanto, em duas semanas.

### Critérios de inclusão e exclusão

Como critérios de inclusão para o convite à família participante, a criança deveria possuir diagnóstico de perda auditiva de qualquer tipo ou grau, sem outras deficiências associadas à deficiência auditiva, fazer uso efetivo de Aparelho de Amplificação Sonora Individual (AASI) e/ou Implante Coclear (IC), ter até seis anos de idade e estar em reabilitação no programa Audição e Linguagem do Centro SUVAG/RN, com frequência igual ou superior a 75%. A família, além da disponibilidade de participar da intervenção intensiva, realizada em 10 dias consecutivos, excetuando o final de semana, deveria ter um dispositivo eletrônico (computador, *tablet* ou *smartphone*) com acesso à internet que permitisse a conexão. Foram estabelecidos critérios de inclusão tendo como foco a população de famílias ouvintes. Desta forma, caso o responsável principal fosse surdo(a), não estaria incluído(a) no estudo, ainda que o mesmo protocolo de intervenção pudesse ser aplicado em famílias surdas, desde que o pesquisador fosse fluente em Língua Brasileira de Sinais (LIBRAS).

Foram elencados como critérios de exclusão: a criança que ainda não estivesse adaptada ao AASI ou IC, usuária da LIBRAS, com frequência inferior a 75% e que possuísse outras deficiências associadas à deficiência auditiva, optando-se assim, estudar um caso sem outras deficiências associadas à perda auditiva. Destacamos, ainda assim, que a intervenção proposta é aplicável a grupos específicos.

Em relação à família, a indisponibilidade de participar da intervenção intensiva, bem como a impossibilidade de acesso à internet foram critérios de exclusão neste estudo. A caracterização da criança e de sua mãe é apresentada nas Tabelas 1 e 2.

**Tabela 1:** Caracterização da criança participante da pesquisa

**Criança**	**Idade (a/m)**	**Gênero**	**IA (anos/meses)**	**Grau da PASN**		**Etiologia da PA**	**Adaptação**	**Tipo de dispositivo**		**Categorias de Audição e Linguagem**	
				**OD**	**OE**			**OD**	**OE**	**CA**	**CL**
C0	5a2m	feminino	3a5m	Profundo	Profundo	Idiopática	Bilateral	IC	AT	5	4

Fonte: próprio autor

**Legenda:** C0 = Criança 0; a/m = anos e meses; IA = Idade auditiva; PA = Perda Auditiva; PASN = Perda Auditiva Sensorioneural de acordo com OMS 2014; OD = Orelha direita; OE = Orelha esquerda; IC = Implante Coclear; AT = Implante Coclear na Assistência Técnica no momento da intervenção; CA = Categoria de Audição; CL = Categoria de Linguagem^(11)^.

**Tabela 2:** Caracterização da família participante da pesquisa

**Família**	**Idade (a/m)**	**NE**	**Ocupação**	**Renda**	**Auxílios**	**RAF**		**EAR**		**EEP**	
F0	34a9m	EMC	Manicure	1 salário-mínimo	não possui	65	67	33	37	26	24

Fonte: próprio autor

**Legenda:** F0 = Família 0; a/m = anos e meses; NE = Nível de Escolaridade; EMC = Ensino Médio Completo; RAF = Recursos do Ambiente Familiar; EAR = Escala de Autoestima de Rosenberg; EEP = Escala de Estresse Parental

### Caracterização do caso e instrumentos de avaliação

Para a avaliação da criança, realizada de forma remota, foram utilizadas as Categorias de Audição e de Linguagem^(11)^ cujos escores foram obtidos a partir da observação do fonoaudiólogo responsável na equipe de reabilitação do serviço e foi aplicado o formulário da família dos Indicadores de Performance Funcional Auditiva Brasileiro - Versão Reduzida (FAPI-r). A versão reduzida do FAPI-r é composta por um formulário para o fonoaudiólogo para aplicação com a criança, constando de 25 itens, e outro para a família com 15 itens, ambos organizados em 15 seções nas quais o familiar informa a frequência com que os comportamentos auditivos são observados em casa, abrangendo as habilidades de consciência sonora, feedback auditivo, discriminação auditiva e reconhecimento, compreensão auditiva, memória auditiva de curto prazo e processamento auditivo linguístico^(12)^.

Na aplicação do FAPI-r (versão família) observou-se que no momento pré-intervenção, de acordo com as observações da mãe, a criança já tinha adquirido as habilidades de consciência sonora (100%), *feedback* auditivo (100%) e estava em processo de aquisição das habilidades de discriminação auditiva e reconhecimento (75%), compreensão auditiva (75%), memória auditiva de curto prazo (66,7%) e processamento auditivo linguístico (75%). No momento pós-intervenção, houve modificação nas pontuações das habilidades de discriminação auditiva e reconhecimento (100%) e compreensão auditiva (100%). As demais permaneceram com as mesmas pontuações. Embora não se possa afirmar que tal mudança decorreu do processo de intervenção realizado, observou-se que as questões em que a mãe relatou melhora nas habilidades auditivas referiram-se a: discriminação da intenção comunicativa das declarações, identificação de elementos críticos em frases (dois e três elementos) e em história infantil, indicando que o processo dialógico enriquecido pela intervenção proposta pode ter influenciado a percepção da mãe e sua maior atenção a estas habilidades durante as interações com a filha.

Para a avaliação da mãe, foram aplicados os seguintes protocolos:

1. Escala de Estresse Parental (EEP) - Instrumento de autorrelato, distribuído em 18 itens representando quatro fatores (recompensas dos pais, estressores dos pais, falta de controle e satisfação parental) respondidos em escala *Likert* (zero a cinco) desenvolvido com o objetivo de mensurar o nível de estresse vivenciado por pais e mães de filhos menores de 18 anos. A pontuação total pode variar entre 0 e 72 pontos, de forma que quanto mais elevado o escore, maior o estresse parental^(13)^.
2. Escala de Autoestima de Rosenberg (EAR) - instrumento com dez sentenças fechadas que propõe avaliar de forma global a atitude positiva ou negativa do indivíduo em relação a si mesmo, respondidos em escala *Likert (um a quatro)*, variando entre “concordo totalmente” e “discordo totalmente”. A pontuação pode oscilar entre 10 a 40 sendo que quanto maior o escore obtido na escala, maior o nível de autoestima do indivíduo^(14)^.
3. Inventário de Recursos do Ambiente Familiar (RAF): roteiro aplicado sob forma de entrevista semi-estruturada para a caracterização dos recursos materiais e das rotinas familiares. A soma da pontuação dos itens assinalados nos quesitos 1 a 7 resultam em sua pontuação bruta, já os tópicos 8, 9 e 10, possuem pontuação específica indicada no formulário^(15)^.

Vale ressaltar que embora tenham sido aplicados tais protocolos para a caracterização e acompanhamento da mãe, neste estudo piloto não tivemos como objetivo a verificação do efeito da intervenção nas medidas de recursos do ambiente familiar, estresse parental e de auto-estima da mãe, considerando a característica específica da intervenção, seu curto prazo e a complexidade das variáveis analisadas.

Observou-se que na avaliação de estresse parental, comparando os escores pré e pós-intervenção, houve discreta diminuição da pontuação, mas na avaliação pré, já apresentava baixos níveis de estresse parental.

A EAR, medida pela somatória dos itens, é classificada nas categorias de autoestima: baixa, média e alta. A mãe, desde a avaliação pré-intervenção possuía uma autoestima elevada, pontuando com valores acima de 30 em ambos os cenários.

Destaca-se, pois, que trata-se de um caso em que as características da mãe (escolaridade, estresse parental e auto-estima) foram positivas para o trabalho terapêutico. É importante, portanto, que tais medidas sejam consideradas na avaliação das famílias para a melhor compreensão das necessidades de cada família para que condutas mais assertivas sejam adotadas.

Sobre o ambiente familiar, observou-se, pelo RAF, uma visão dos recursos, características e comportamentos dispostos em casa, com discreto aumento dos exemplos dados pela responsável. A mãe descreveu a proximidade da família na realização das atividades compartilhadas dentro e fora do ambiente do lar e com regularidade. Foi percebido mudança nas exemplificações do item 4, na qual no momento pré intervenção a mãe listou o *“Brincar, Assistir a filmes, Assistir a programas infantis na TV, Ler livros, revistas“* e após a intervenção adicionou/substituiu pelos itens *“Brincar, Assistir a filmes, Ouvir as estórias da criança; conversar sobre os assuntos que ela traz.* No item 8, a mãe referenciou a si mesma em todas as situações expostas, para além da cuidadora principal, uma agente que conduz as atividades estimuladoras. A disponibilidade de recursos físicos como oferta de brinquedos, brincadeiras, revistas e livros se mantiveram no pré e pós-intervenção.

Há, portanto, a partir do relato da mãe, uma reorientação da atenção dela, no pós-intervenção, para ouvir mais o que a criança traz na rotina diária, bem como a percepção de seu empoderamento como um agente que coopera com a aprendizagem de sua filha, a partir das atividades do dia a dia.

Além destas avaliações, todos os 11 vídeos de interação registrados no período de 9 a 21 de janeiro de 2022 (excetuando os finais de semana) foram analisados a partir do Instrumento de Análise de Interação Família-Criança (adaptado pelos pesquisadores de Cole^(16)^) por 3 juízes independentes, sendo dois deles cegos. Os juízes possuem formação em fonoaudiologia, com experiência na reabilitação auditiva superior a 4 anos e familiaridade com a utilização da ferramenta na prática clínica. O Instrumento de Análise de Interação Família-Criança é constituído de uma escala observacional dos comportamentos comunicativos na interação dos pais ouvintes com seus filhos com deficiência auditiva, na qual a frequência dos comportamentos é pontuada em uma Escala do tipo *Likert,* de 1 a 7, sendo o comportamento de menor frequência “Não vi” e o de maior frequência “Vi sempre”, e ainda campos de “não tenho certeza” ou “não se aplica”, nas dimensões emocionais e comportamentais da criança e do cuidador principal. O instrumento também contém a análise geral da competência comunicativa do cuidador, pontuada em escala de: 0 (não mostra competência), 1 (mostra um pouco de competência), 2 (mostra uma boa competência) ou 3 (mostra uma competência excelente). A concordância entre os juízes apresentou-se quase-perfeita, com Kappa de 0,79 e 91% de concordância (p ≤ 0,007), conforme a Tabela 3.

**Tabela 3:** Concordância entre os juízes

**Método**	**Cohen's Kappa**
Vídeos	11
Juízes	2
Concordância%	91
Kappa	0,792
z	2,69
p-valor	0,007

Fonte: próprio autor

### Intervenções concomitantes ao estudo

Paralelamente às sessões de teleintervenção com VF deste estudo, a criança e sua mãe continuaram participando dos atendimentos em grupo e individuais do programa de Audição e Linguagem ofertado pelo serviço, neste momento, também de forma remota devido à pandemia, a saber: uma vez na semana para os atendimentos fonoaudiológicos individuais com a família e a criança de acordo com o Método Aurioral, três atendimentos semanais em grupo, dois voltados para estimulação auditiva e um para as necessidades familiares. A duração de cada um destes atendimentos era de 30 minutos.

### Procedimentos da teleintervenção com *Videofeedback*

A teleintervenção com VF baseou-se em modelo já testado com famílias de crianças com deficiência auditiva^(5,6)^ e foi realizada em 10 sessões síncronas de 50 minutos, com enquadre individual.

Para a realização dos procedimentos com a família, a pesquisadora utilizou um computador com *webcam* e fones de ouvido e conexão de internet banda larga de fibra óptica, cabeada, com 400 *megabytes* de velocidade de conexão. O ambiente da teleconsulta seguiu todas as normativas da resolução CFFa nº 580/2020^(9)^, realizado em plataforma segura, de forma sigilosa.

Para a operacionalização da teleintervenção foi necessário o treinamento prévio da mãe para a sua familiarização com a plataforma de videochamada *GSuite*, além da preparação para a gravação e upload dos vídeos via *Drive GSuite*, que compreendeu a primeira das 13 sessões. Os vídeos de interação foram registrados pela própria mãe, em celular próprio, com duração média de dez minutos por vídeo e em seguida, estes eram compartilhados com a pesquisadora pelo armazenamento em nuvem. Após a sinalização de envio, a sessão de VF era agendada.

É importante ressaltar que o ambiente de registro dos vídeos era natural, familiar à criança e com os recursos disponíveis na própria casa. A instrução para a mãe foi a de que interagisse com a criança a partir de situações naturais da casa, como na sua rotina.

A pesquisadora principal analisou cada vídeo de interação e procedeu com o planejamento das sessões, destacando sempre 3 pontos positivos observados na interação e preparando orientações que se adequassem ao aperfeiçoamento da interação comunicativa da mãe com a filha. O vídeo de interação com a seleção dos micro-momentos positivos era exibido para a mãe pela função de compartilhamento de tela do *Google Meet*. Durante as sessões, que ocorrem de maneira dialogada, a mãe era levada a refletir sobre o clipe apresentado e eram discutidos os pontos positivos de cada clipe e orientações eram dadas, no sentido de aperfeiçoar a comunicação.

Todos os dados analisados foram inseridos em uma planilha *Excel®* para sua análise descritiva e qualitativa, a partir das observações realizadas ao final do protocolo adaptado^(16)^.

A análise inferencial dos dados apresentada neste manuscrito, foi realizada com a aplicação método estatístico JT^(17)^ o qual permite analisar a mudança confiável e a significância clínica por meio da verificação da confiabilidade das mudanças obtidas entre os escores pré e pós-intervenção, com a finalidade de identificar se as mudanças, mesmo em estudos com único ou poucos participantes, são de caráter confiável e se são relevantes clinicamente, investigando o Índice de Mudança Confiável – IMC e a Significância Clínica das mudanças.

Os resultados apresentados a seguir compreendem a análise dos vídeos realizada pelos juízes cegos, sendo destacadas as variáveis dos comportamentos de competência comunicativa geral da mãe e da linguagem receptiva e expressiva da criança.

### Resultados

Sobre o processo de intervenção, notou-se que houve evolução da competência geral da comunicação da mãe ao longo das sessões, sendo que a mesma saiu de escores de 1 para 2 (de pouca competência para boa competência), até o oitavo vídeo manteve-se entre os escores 2 e 3 (com exceção do vídeo 7, em que foi notada leve queda de seu desempenho na interação) e a partir do nono vídeo, a mãe que tinha escores de boa competência comunicativa (2), foi avaliada pelos 2 juízes e pela pesquisadora como tendo excelente competência geral na comunicação durante a interação, o que indica que 8 sessões foram necessárias para que a mãe atingisse o nível máximo de desempenho na interação de acordo com o instrumento de análise empregado, conforme apresentado nas Figuras 1 e 2.

**Figura 1:** Desempenho Comunicativo Geral da Mãe ao longo do processo de teleintervenção guiada por *videofeedback*. **Fonte:** Próprio autor

**Legenda:** V1 a V11: Vídeos 1 a 11. 0: não demonstra competência comunicativa; 1:demonstra pouca competência comunicativa; 2: demonstra boa competência comunicativa; 3: demonstra excelente competência comunicativa

**Figura 2:** Índice de mudança confiável da variável “Competência Geral da Mãe na Interação” pré e pós sessões de teleintervenção guiada por *videofeedback*. **Fonte:** Próprio autor

Sobre o desempenho da criança, conforme apresentado nas Figuras 3, 4 e 5, observou-se que houve melhora na linguagem receptiva e expressiva, sendo que a intervenção produziu mudança positiva confiável (acima do intervalo de confiança) para as variáveis estudadas do comportamento da criança: “Vocabulário Receptivo” e Vocabulário Expressivo”.

**Figura 3:** Comportamentos da Criança: Linguagem Receptiva e Expressiva. **Fonte:** Próprio autor

**Figura 4:** Índice de mudança confiável da variável Linguagem Receptiva pré e pós sessões de teleintervenção guiada por *videofeedback*. **Fonte:** Próprio autor

**Figura 5:** Índice de mudança confiável da variável Linguagem Expressiva pré e pós sessões de teleintervenção guiada por *videofeedback*. **Fonte:** Próprio autor

## DISCUSSÃO

A competência comunicativa do cuidador principal da criança com deficiência auditiva tem se demonstrado um fator importante para o seu desenvolvimento de audição e linguagem^(8)^ e, portanto, intervenções que otimizem a interação dialógica entre as famílias e cuidadores com estas crianças são importantes e passíveis de serem implementadas em serviços de reabilitação auditiva infantil, preferencialmente de forma precoce^(2-7)^.

Em nosso estudo de caso observamos que houve melhora progressiva da competência de comunicação da mãe ao longo das sessões e que a partir da oitava sessão (nono vídeo) observou-se um salto qualitativo importante nesta variável. Por meio do Método JT^(17)^, foi possível demonstrar, pois, simultaneamente a confiabilidade das mudanças e a significância clínica, neste caso, observada na mãe (Figura 2).

Assim, a mãe apresentou escores distintos pré e pós intervenção, atingindo um desempenho compatível com a de uma população não clínica, com enquadre na classificação de “recuperação” sugerida pelos autores^(17)^ quando ambos critérios são atingidos, dado que corrobora com a literatura sobre o potencial da ferramenta de VF no aperfeiçoamento do comportamento comunicativo e da interação entre cuidadores ouvintes e seus filhos(as) com deficiência auditiva^(2,5,6)^.

Observou-se durante as teleconsultas que a oportunidade de ser diretamente o agente estimulador da criança, ainda que sob orientação remota do fonoaudiólogo, promoveu uma mudança importante e necessária no papel desta mãe em relação à sua filha, o que é confirmado por estudos que empregaram a telefonoaudiologia no atendimento à população de famílias de crianças com deficiência auditiva^(7)^.

Deste modo, ao considerarmos o número de horas em que a criança com deficiência auditiva regularmente está em casa, e - dependendo da faixa etária - no ambiente escolar, justifica-se o emprego de métodos que direcionam a atenção da família e/ou cuidadores da criança a otimizar suas interações diárias, tornar-se um agente otimizador das possibilidades de aprendizado cotidiano destas crianças^(2-8)^.

Sobre o efeito das intervenções parentais no desenvolvimento da linguagem da criança com deficiência auditiva, alguns estudos apontam melhoras significativas^(2-4)^, no entanto, evidências mais robustas são necessárias para afirmar se o efeito das intervenções às famílias mantém-se ao longo do tempo, assim como o seu impacto no desempenho da criança longitudinalmente.

Para a criança participante deste estudo, a partir da análise do Índice de Mudança Confiável (IMC)^(17)^ calculado por meio dos valores do instrumento de avaliação aplicado, foi considerado índice confiável e positivo quando os valores atingiram números superiores à 1,96 e, desta forma, podemos inferir que a intervenção produziu mudança positiva confiável (acima do intervalo de confiança) para as variáveis estudadas do comportamento da criança: “Vocabulário Receptivo” (Figura 4) e “Vocabulário Expressivo” (Figura 5).

No entanto, não houve significância clínica para ambas as medidas, ou seja, houve melhora nos comportamentos observados na criança em sua linguagem receptiva e expressiva, porém não o suficiente para posicioná-la na população não-clínica. Este dado era esperado, já que as necessidades de desenvolvimento da linguagem na criança com deficiência auditiva são amplas^(8)^ e 10 sessões de terapia indireta (focalizada na capacitação da mãe) não seriam esperadas como suficientes para atingir todas as necessidades de intervenção fonoaudiológica específicas para o desenvolvimento linguístico da criança. Ainda assim, houve melhora confiável (mudança positiva confiável), sendo a participante categorizada em “melhorado” (atingiu o IMC mas não a significância clínica)^(17)^.

Por não se tratar de um ambiente clínico e controlado, algumas variáveis como a não interferência de outros familiares, ruídos externos e dificuldade de posicionamento da câmera foram percebidas durante as sessões. Em contrapartida, foi observada a espontaneidade na interação entre a mãe e a criança, a disponibilidade de recursos materiais a partir da rotina e também a ambientação confortável da criança em situação não clínica. Ao analisar as tentativas de comunicação da criança, do adulto e a troca de turnos de conversação, pode-se compreender o real ambiente linguístico desta criança. Tal análise, também possibilitada por recursos tecnológicos como o LENA (*Language ENviromental Analysis System*)^(8)^, pode vir a aperfeiçoar intervenções como a teleintervenção guiada por VF.

Diante o exposto, a partir deste estudo de caso, destaca-se que há a necessidade de se empregar esta metodologia em estudos experimentais randomizados e com n amostral adequado para se estimar os efeitos deste modelo de capacitação parental, de modo a otimizar os esforços para o sucesso terapêutico na população de crianças com deficiência auditiva.

## COMENTÁRIOS FINAIS

Dada a escassez de pesquisas sobre programas com VF e sua aplicabilidade na reabilitação auditiva, este estudo mostra-se relevante por contribuir com a produção de evidências sobre a efetividade da teleintervenção por VF e ampliar a possibilidade de implementação de programas utilizando esta ferramenta.

Ainda que se trate de caso único, a análise com o método JT permitiu a verificação da efetividade da intervenção, medida pela mudança confiável dos comportamentos de interação da mãe com a filha e da criança em relação à linguagem receptiva e expressiva, com mudança clínica significativa nos comportamentos comunicativos da mãe.

Por tratar-se de um estudo de caso único ainda não é possível generalizar tais resultados, sendo necessário o desenvolvimento de outras pesquisas com maior robustez metodológica e investigação das garantias de manutenção de resultados com a intervenção, sendo esta a principal limitação deste estudo.

A continuidade das pesquisas na temática da capacitação parental para famílias de crianças com deficiência auditiva é relevante no Brasil, dada a diversidade de nossa cultura e das situações de vulnerabilidade que podem repercutir na necessidade de desenvolvimento de modelos específicos de capacitação para a nossa população.
